# Sex as a strategy against rapidly evolving parasites

**DOI:** 10.1098/rspb.2016.2226

**Published:** 2016-12-28

**Authors:** Stuart K. J. R. Auld, Shona K. Tinkler, Matthew C. Tinsley

**Affiliations:** Division of Biological and Environmental Sciences, University of Stirling, Room 3B164, Cottrell Building, Stirling, Stirlingshire FK9 4LA, UK

**Keywords:** evolution of sex, Red Queen, parasitism, coevolution

## Abstract

Why is sex ubiquitous when asexual reproduction is much less costly? Sex disrupts coadapted gene complexes; it also causes costs associated with mate finding and the production of males who do not themselves bear offspring. Theory predicts parasites select for host sex, because genetically variable offspring can escape infection from parasites adapted to infect the previous generations. We examine this using a facultative sexual crustacean, *Daphnia magna,* and its sterilizing bacterial parasite, *Pasteuria ramosa*. We obtained sexually and asexually produced offspring from wild-caught hosts and exposed them to contemporary parasites or parasites isolated from the same population one year later. We found rapid parasite adaptation to replicate within asexual but not sexual offspring. Moreover, sexually produced offspring were twice as resistant to infection as asexuals when exposed to parasites that had coevolved alongside their parents (i.e. the year two parasite). This fulfils the requirement that the benefits of sex must be both large and rapid for sex to be favoured by selection.

## 1. Background

One of the great paradoxes of biology is that sex is the dominant mode of reproduction when asexual reproduction is much less costly [1–3]. When all else is equal, asexual populations have a higher effective birth rate than sexual populations, because asexuals do not produce males [3], benefit from coadapted gene complexes that sexual recombination would break apart, and avoid the many and varied costs associated with securing a suitable mate. Asexuals should rapidly outcompete their sexual counterparts [4], yet in reality, sex is the dominant mode of reproduction among Eukaryotes [5]. All else is clearly not equal.

Many hypotheses have been put forward to explain why sex dominates over asex [5,6]. Chief among them is the Red Queen hypothesis, which states that parasite-mediated selection is strongest against common contemporary host genotypes [7–10]. Red Queen dynamics may favour sex over asex, because sex (specifically, recombination associated with sex) can recycle alleles in such a way to continually generate novel resistance genotypes on which selection can act, thus maintaining host fitness despite endlessly evolving virulent parasite populations [11,12]. There is compelling evidence that parasites can generate this strong selective force: previous work has demonstrated that parasites adapt to locally common host genotypes in time or space [13–16].

A productive method for testing whether parasitism favours sex over asex involves comparing parasite resistance of obligately asexual and sexual host lineages of a particular host species [16–18]. Such studies have effectively demonstrated that obligate sexual lineages can outcompete obligate asexual lineages in the face of parasitism. A complementary, though more rarely used, approach that provides a direct test of the benefits of sex over asex involves using facultative sexual organisms to compare the parasite susceptibility of sexual and asexual offspring from a single parent. Kelley *et al.* [19] demonstrated that sexually produced offspring of the grass, *Anthoxanthum odoratum* had higher reproductive rates than their asexually produced counterparts when planted near the maternal plant in a biologically realistic scenario; a subsequent study found that such differences could be explained by differences in susceptibility to a virus [20]. Here, we directly test the role of host reproductive mode on parasite resistance using a facultative sexual host, the crustacean *Daphnia magna*, and its sterilizing bacterial parasite, *Pasteuria ramosa*.

We collected healthy wild *D. magna* that were carrying sexual eggs, and *Pasteuria* isolates from a natural pond population. By allowing the healthy *Daphnia* to revert to asexual reproduction after releasing their sexual eggs, and by hatching the sexuals and maintaining them in a clonal state, we were able to take a genetic snapshot of both the maternal (asexual) and offspring (sexual) generation (see also [21], who used this method to examine *Daphnia* inbreeding in the wild and in the laboratory). To test the effect of rapid parasite evolution on offspring fitness, we collected additional *Pasteuria* isolates from the same population the following year. Ordinarily, in UK populations, *Daphnia* can only pass sexual offspring on into the next year, because only sexual eggs can undergo diapause and withstand winter conditions. However, this simple time-shift experiment allowed us to simulate host strategies where either asexually produced eggs can survive to hatch in the subsequent year, or sexually produced eggs hatch immediately and face a non-evolved parasite population. Indeed, previous time-shift experiments have taught us much about the nature of host−parasite coevolution [22]. Here, we recorded the two principal infection characteristics: proportion of hosts infected and *Pasteuria* transmission spore density per infected host. By analysing both the overall trends and underlying family-level genetic correlations, we were able to add to previous work examining the relative fitness of sexually and asexually produced offspring [19,23]. Collectively, our findings demonstrate that the parasite population evolved rapidly in the field, whereas our laboratory experiments indicate temporal changes in parasite-mediated selection on host genotypes, favouring host sex over asex.

## 2. Methods

### (a) Study system

In natural populations, sexually produced *Daphnia* eggs hatch in the spring, and develop into adults that reproduce asexually. *Daphnia* later undergo sex as population density (and often parasite prevalence) peaks [24,25], then revert to asexual reproduction. The sexually produced eggs are deposited in the sediment and remain in a state of diapause; these diapausing sexual eggs hatch in the next or subsequent years to face a parasite population shaped by coevolution with previous host generations [14,26]*. Daphnia* are orally infected by *P. ramosa*, a sterilizing bacterial parasite, throughout the season (lasting from spring to early winter: [26]). The likelihood of infection depends on genotypic specificity, i.e. the precise combination of *Daphnia* and *Pasteuria* spore genotypes [27]. When infection does occur, it leads to complete and rapid host sterilization, and, after a period of within-host growth, *Pasteuria* transmission spores are released from dead hosts [28].

### (b) Sampling and experimental protocol

In June 2013 (year one), we collected 100 *P. ramosa-*infected *D. magna*, and 52 healthy female *Daphnia-*carrying ephippia (the melanized case containing one or two sexually produced eggs) from a natural population at Kaimes Farm, Leitholm, Scottish Borders, UK (2°20′43″W, 55°42′15″N; see [26]). One year later (June 2014; year two), we collected another 100 *Pasteuria-*infected *Daphnia*. Individual infected *Daphnia* contained 0.4–6.1 million *Pasteuria* transmission spores.

**Figure 1.**
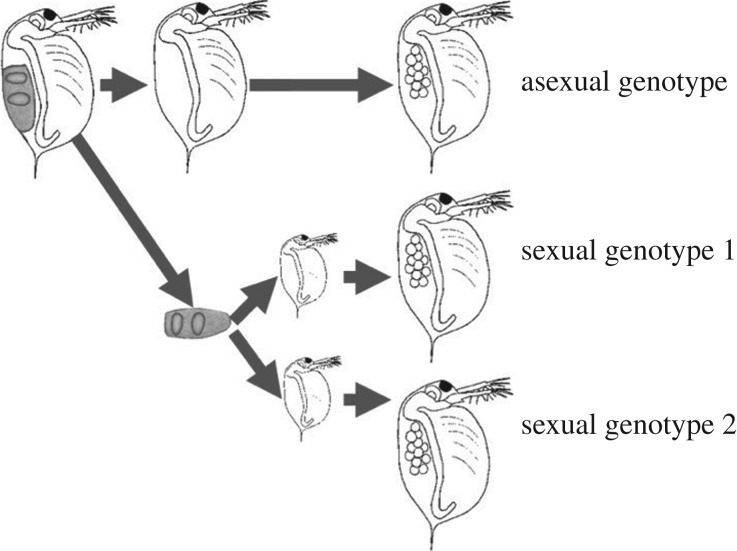
Method for establishing ‘families’ of asexual and sexual *Daphnia* genotypes (adapted from [21]). *Daphnia-*carrying sexual eggs were collected from the wild and kept individually. Once the sexual eggs were released, they were hatched and all genotypes (asexual and sexual) were maintained clonally in the laboratory.

Once in the laboratory, we homogenized infected females, pooled the resulting spore suspensions according to collection year and stored them at −20°C. Healthy females were kept individually in the laboratory; once they released their ephippia, the mothers returned to asexual reproduction (these offspring were used to establish the asexual genotype line for each family). We washed the ephippia in 10% bleach, and then kept them individually in 1 ml artificial medium [29] modified using one-twentieth of the recommended selenium dioxide (SeO_2_) concentration [30]. Ephippia were exposed to natural sunlight on a windowsill and were monitored daily for hatching. Twenty-one of the 54 ephippia hatched (10 ephippia yielded one offspring, 11 ephippia yielded two offspring). There were 21 *Daphnia ‘*families’: 10 families consisted of one asexual and one sexual genotype; 11 families consisted of one asexual and two sexual genotypes (figure 1).

We maintained eight independent replicates for each *Daphnia* genotype for three generations to minimize variation in condition that could otherwise have resulted from environmental, maternal, or epigenetic effects. Animals were kept in jars containing 200 ml of modified artificial medium and fed 1.0 ABS *Chlorella vulgaris* algal cells per *Daphnia* per day (ABS is the optical absorbance of 650 nm white light by the *Chlorella* culture). We refreshed their medium three times per week. There were five *Daphnia* per jar and jars were incubated at 20°C on a 12 light (L) : 12 dark (D) light cycle. The second-clutch neonates from the third generation were used for the experiment.

We allocated neonates from each maternal family genotype to one of two parasite infection treatments (year one or year two parasites), following a split clutch design. There were eight replicates per host genotype per parasite treatment. Each experimental replicate consisted of eight hosts kept in 200 ml of artificial medium. Parasite exposures were conducted as follows: replicates received 1 × 10^5^ *Pasteuria* spores from either year one or year two isolates. Replicates were stirred daily and fed low food (0.5 ABS *Chlorella* per *Daphnia* per day) for the duration of parasite exposure to increase spore uptake by hosts. Parasite exposure lasted 5 days. After the parasite exposure period, replicates were changed into fresh medium and given standard food (1.0 ABS *Chlorella* per *Daphnia* per day). Replicates received fresh medium three times per week and the experiment lasted for 30 days. We visually scored the proportion of infected hosts on experimental day 20. On day 30, *Daphnia* from each replicate were pooled, homogenized with 100 µl of ddH_2_O, and three independent counts were made from the resulting suspension using a Neubauer (improved) counting chamber (0.0025 mm^2^ × 0.1 mm depth).

### (c) Statistical analyses

All statistical analyses were performed using the MCMCglmm (Markov Chain Monte Carlo generalized linear mixed model) package in R 3.0.2 [31,32] (all data are deposited in Dryad doi:10.5061/dryad.nk27k). This approach to linear mixed-effects model analysis allowed us to estimate confidence intervals on the magnitude of our random effects and on the genetic covariances across different treatment conditions. We fitted three models. First, we ensured the sampled asexual genotypes were genetically diverse by quantifying the proportion of the variance in host resistance to year one parasites that was explained by the identity of the asexual genotypes (model 1). Next, using data for both sexual and asexual genotypes and parasite isolates from both years, we tested the effects of reproductive mode (asexual or sexual) and year of parasite collection (year one or year two) on both the proportion of infected hosts (model 2) and the density of parasite spores in infected hosts (model 3). For all three models, we used Bayesian Markov chain Monte Carlo techniques to estimate the posterior mode and 95% credible intervals (CIs) for the fixed effects of host reproductive mode, parasite year, and their interaction.

For model code, see the electronic supplementary material. In model 1, we fitted host genotype as a random effect to the infection risk data for the asexual genotypes and year one parasite isolate only (the model contained no fixed effects). For models 2 and 3, we fitted random effects for host genotype, as well as for host family (each family comprised one asexual and up to two sexually derived sister genotypes). For the family random effect, we specified an unstructured variance−covariance matrix with an interaction between family, reproductive mode, and parasite year. This unstructured matrix allowed for heterogeneity in the between-family variance across each of the four reproductive mode-by-parasite year combinations, as well as covariance between the family means under each of these four treatments. We allowed the residual variance in infection to differ between parasite isolates from the two years. The data for the proportion of infected hosts in each jar were logit-transformed (models 1 and 2) and the spore densities were log-transformed (model 3) to achieve a Gaussian distribution. The MCMCglmm models had parameter-expanded priors and were run for 1 300 000 iterations, with a burn-in of 300 000, sampling each 250th iteration. Autocorrelation was low among consecutive thinned observations, variance terms, and fixed effects (all less than 0.04). We tested for, and found, convergence using the Heidelberger and Welch stationarity diagnostic [33]. Further, Gelman−Rubin diagnostics [34] demonstrated that multiple model runs converged on the same posterior distribution (three runs yielded a multivariate potential scale reduction factor of 1.00).

## 3. Results

### (a) Host sex and infection risk

**Figure 2.**
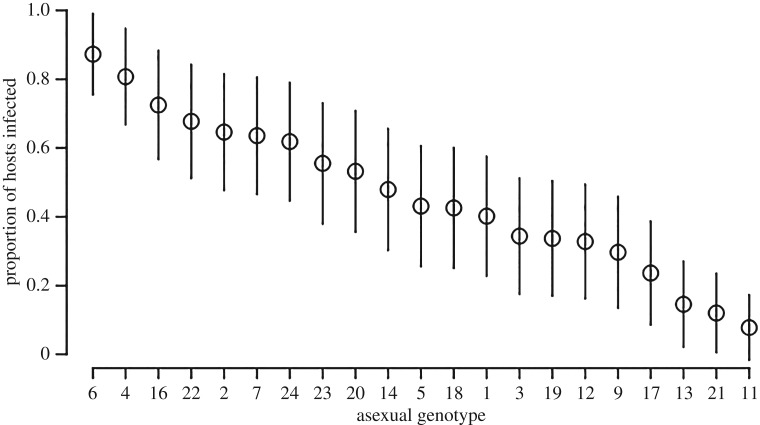
Variation in infection risk across the 21 original wild-collected asexual host genotypes when exposed to year one parasites (mean ± binomial errors).

The asexual genotypes we sampled from the wild differed in their resistance to parasitism (figure 2): when challenged with the year one parasite isolate, the proportion of infected hosts varied from 0.08 to 0.87, and differences between host genotypes explained a high proportion (0.59 ± 0.41–0.75 95% CIs) of the random effect variance.

**Figure 3.**
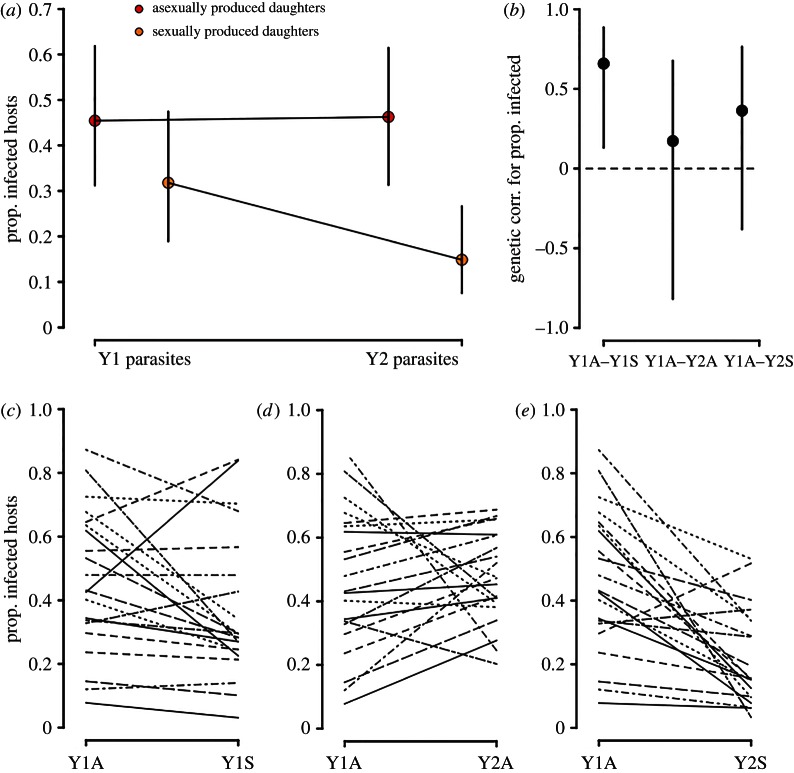
Effect of host reproductive mode and parasite year on infection risk. (*a*) Proportion of infected hosts according to reproductive mode and parasite year (posterior mode ± 95% credible intervals (CIs)). (*b*) Genetic correlation coefficients for proportion infected hosts (±95% CIs) between asexual and sexual offspring when exposed to year one parasites (Y1A–Y1S), between asexual offspring exposed to year one and year two parasites (Y1A–Y2A), and when asexual offspring are exposed to year one parasites and sexual offspring are exposed to year two parasites (Y1A–Y2S). Genetic correlations are significant when the 95% CIs do not overlap zero (see dashed line). (*c*–*e*) Family-level phenotypic associations between treatment categories. (Online version in colour.)

When hosts were challenged with the year one parasite, the difference in resistance between asexual and sexual offspring was small and non-significant (figure 3*a*: reproductive mode effect *P*_MCMC_ = 0.09; electronic supplementary material, table S1). Crucially, when offspring were exposed to year two parasites, which had coevolved alongside their parents in the previous year, the asexually produced genotypes had low resistance to infection (46% infected), whereas sexually produced offspring were over twice as resistant (15% infected, figure 3*a*: reproductive mode × parasite year interaction *P*_MCMC_ = 0.01; electronic supplementary material, table S1). These changes in infection risk were specifically associated with host sex; they were not driven by a general change in parasite infectivity across years (parasite year effect *P*_MCMC_ = 0.91; electronic supplementary material, table S1).

We also found that infection risk was highly correlated between the sexual and asexual genotypes of each family when challenged with year one parasites (figure 3*b*,*c*), demonstrating a strong heritable component to parasite resistance. By contrast, there was no correlation in resistance between asexuals exposed to year one parasites and asexuals exposed to year two parasites (i.e. when the host genetic background is identical but the parasite samples differ); this confirms the wild parasite population rapidly evolved over the course of a single year (figure 3*b*,*d*). The genetic correlation that reflects natural host reproduction (between asexuals exposed to year one parasites and sexuals exposed to year two parasites) was also absent; moreover, the rank order of host family infection risk changed across parasite isolates (figure 3*c*,*e*), demonstrating that parasite evolution exerts temporally shifting selection on the host population over very short timescales.

### (b) Host sex and parasite within-host growth

**Figure 4.**
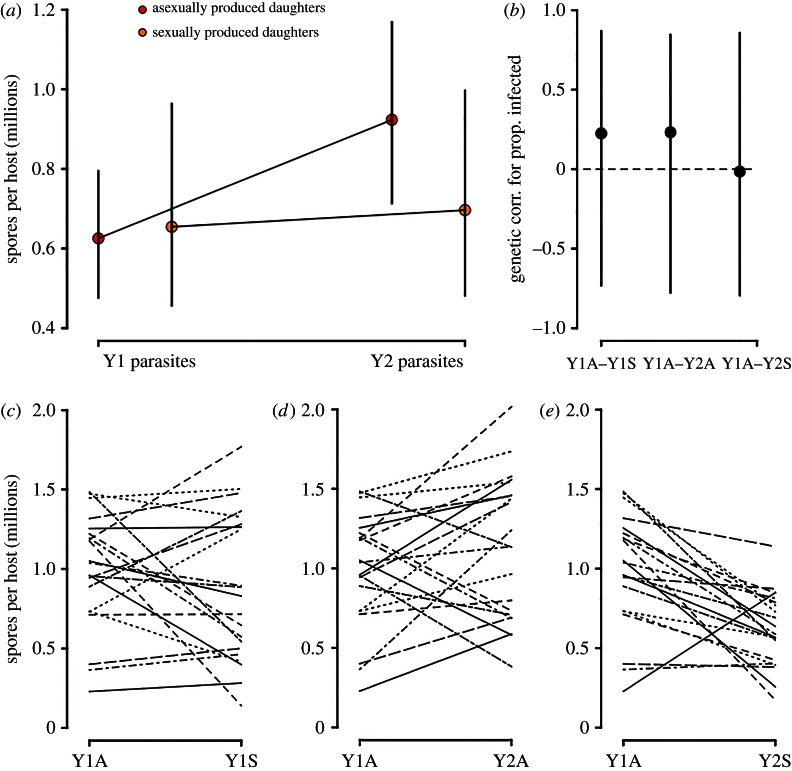
Effect of host reproductive mode and parasite year on parasite burdens. (*a*) Spore densities in infected hosts according to reproductive mode and parasite year (posterior mode ± 95% CIs). (*b*) Genetic correlation coefficients for spore densities (±95% CIs) between asexual and sexual offspring when exposed to year one parasites (Y1A–Y1S), between asexual offspring exposed to year one and year two parasites (Y1A–Y2A), and when asexual offspring are exposed to year one parasites and sexual offspring are exposed to year two parasites (Y1A–Y2S). (*c–e*) Family-level phenotypic associations between treatment categories. (Online version in colour.)

After just one year of parasite evolution in the wild, parasite within-host growth was 48% greater within asexually derived host offspring (figure 4*a*: parasite year effect *P*_MCMC_ = 0.002; electronic supplementary material, table S1). Sexually produced daughters were not intrinsically better than asexual daughters at resisting the year one parasite within-host growth (reproductive mode effect *P*_MCMC_ = 0.81; electronic supplementary material, table S1). However, we found some evidence that sexually produced daughters were better at resisting within-host growth of the year two parasite (which has an immediate coevolutionary history with the maternal host generation): sexual genotypes had half the mean spore burden of their asexual counterparts (the host reproductive mode by parasite year interaction was marginally significant *P*_MCMC_ = 0.07; electronic supplementary material, table S1). Further analysis revealed there were no significant genetic correlations for parasite within-host growth comparing across parasite isolates and host reproductive modes (figure 4*b*–*e*).

## 4. Discussion

Studies that directly test the effect of reproductive mode on host resistance to parasitism by comparing the sexual and asexual offspring of a single parent are very rare, and are mainly limited to plant−parasite systems. A short-term benefit of sex over asex was found in a grass system [21], which was driven by variation in resistance to a virus [20]. Although a similar study conducted with wild garlic found that sex was not only disadvantageous compared with asex, but also that common genotypes were favoured by selection [23]. Our study used a facultative sexual animal host. The host population we sampled contained a diversity of asexual clones that varied significantly in their parasite resistance (figure 2). We tested whether host sexual reproduction provided an advantage over asexual reproduction for defence against a highly virulent coevolving parasite. Because selection only favours traits that provide an immediate fitness advantage, the benefits of sex must be both large and occur rapidly for it to be favoured over asex [3]. We found a rapid and considerable advantage of sex over asex in terms of host offspring resistance to sterilizing infection; this advantage emerged when host offspring were challenged with field-collected parasites that had coevolved for just one year with the parental host generation (figure 3).

A key advantage in our approach is that we could examine the genetic correlations for infection traits between asexual and sexually produced offspring, i.e. the correlations between resistance in offspring generated by asexual and sexual reproduction when challenged with contemporary or future parasites. This allowed us to test three key hypotheses that are fundamental to a benefit of sex: (i) a heritable component to disease resistance, (ii) rapid parasite evolution, and (iii) temporally shifting parasite-mediated selection on the host population. Heritability of resistance is demonstrated by a significant correlation between resistance in asexual and sexual offspring challenged with year one parasites. Rapid parasite evolution is demonstrated by an absence of a correlation between the resistance of asexual genotypes exposed to year one parasites and the same asexuals exposed to year two parasites. The absence of a correlation occurred, because there was a dramatic change in the susceptibility rank order of asexual genotypes when they were challenged with the different parasite isolates collected only one year apart. This change in genotypic rank order demonstrates that parasite-mediated selection pressures changed between years, consistent with the Red Queen theory.

Theories of sex hypothesize that parasite evolution rapidly erodes the fitness of lineages that reproduce asexually [12]. Contrary to expectations, we did not find that asexuals exposed to year two parasites suffered more infections than asexuals to year one parasites. However, we did find support for this hypothesis in a second infection metric: parasite burdens within infected hosts. There was strong evidence for rapid parasite adaptation to grow within asexually produced offspring that were genetically identical to their mothers (figure 4). These findings convincingly support previous studies that have demonstrated evolution in the *Pasteuria* parasite [26,35]. Further analysis revealed there were no significant genetic correlations for parasite within-host growth comparing across parasite isolates and host reproductive modes (figure 4*b*–*e*). This result was expected, as parasite within-host growth is dependent on the precise combination of co-infecting parasite genotypes and on the order in which these genotypes arrive [36]. This stands in contrast to our findings concerning infection risk, because infection risk depends chiefly on the interactions between host and parasite genotype (i.e. genotypic specificity [27,37]).

The evolution of increased parasite growth on asexually produced genotypes reflects rapid parasite adaptation over the course of a single year. Nevertheless, this adaptation provides the parasite with no benefit for replication within the genetically novel sexual genotypes that exist in the following season (figure 3*a*). Because *Daphnia* undergo sexual reproduction before a peak in the epidemic [25] and sexual eggs hatch in future years, sexually produced offspring ‘opt out’ of parasite-mediated selection in the current year, i.e. they disperse through time. Early season sex means hosts archive genetic variation in resistance for the future in the same way that plant populations often have a seed bank. Importantly, this genetic archiving occurs before parasite-mediated selection has the opportunity to strip genetic variation from the standing host population (figures 3 and 4).

After undergoing sexual reproduction and releasing diapausing (sexual) eggs, *Daphnia* mothers return to reproducing asexually [25]. This return to asex after sexual reproduction may increase the likelihood that some asexually produced daughters successfully overwinter and survive into the next season (provided that the winter is not unduly harsh). However, overwintering asexuals would suffer greater parasite burdens than their sexually derived counterparts and are thus likely to be outcompeted. If obligately asexual genotypes of *D. magna* emerged, they would be a prime target for parasite adaptation and would therefore be rapidly purged from the population (figure 4).

Our hosts and parasites were all isolated from a natural population and the parasite evolution that selected for host sex occurred under natural conditions. Our laboratory infection experiments demonstrate the potential for rapidly evolving and virulent parasites to select for sex in the wild. Other studies have shown parasite-mediated fitness differences between obligate asexual and sexual lineages in the field [16–18]. Such studies can capture host–parasite interactions in truly natural settings, though this comes with the cost that one cannot exclude the possibility that lineages differ for reasons other than reproductive mode. Our approach, which uses a facultative sexual host, enables direct comparisons between individuals that differ only in their reproductive mode. We must, however, acknowledge that sexual and asexual progeny of *Daphnia* would not ordinarily compete in natural populations, because sexually and asexually produced offspring are present in populations at different times. Also, while *Pasteuria* infection prevalences in our experiment are similar to those in the wild, the exposure regime is artificial. Laboratory and field-based approaches are bound by a trade-off between ecological realism and experimental control; they, nevertheless, complement one another and collectively demonstrate that parasites can provide the strong selective force needed to maintain sexual reproduction.

In summary, our study demonstrates a benefit of host sex in the face of a rapidly evolving parasite, which is realized within a very short timescale. This is particularly important, because the parasite is highly virulent [27], which means host resistance is strongly associated with fitness. Our findings therefore support theory that genetic recombination enables unfit mothers to give rise to fit offspring provided that the direction of selection fluctuates rapidly, and are consistent with the Red Queen hypothesis [12].

## Supplementary Material

Sex as a strategy against rapidly evolving parasites, Auld et al - supplementary information

## Data accessibility

Data are available from Dryad http://dx.doi.org/10.5061/dryad.nk27k [38] and associated R code is in the supplementary material.
