# Supplementary material for: Sex as a strategy against rapidly evolving parasites
Source: Proc Biol Sci. 2016 Dec 28;283(1845):20162226. doi: 10.1098/rspb.2016.2226 (PMC5204169; doi:10.1098/rspb.2016.2226)
Supplement: Sex as a strategy against rapidly evolving parasites, Auld et al - supplementary information [file rspb20162226supp1.pdf]

Model 1 tests for significant genetic variation for proportion of hosts infected among asexual genotypes exposed to year 1 parasites only. See below for the R code for the prior and model

```
block<-factor(block)
genotype<-factor(genotype)

prior1 <- list(R=list(V=diag(1)*1, nu=0.002),
               G=list(G1=list(V= diag(1), nu=2,
 alpha.mu=rep(0,1), alpha.V=diag(1))))
prior5

m1<-MCMCglmm(logit(p.inf) ~  block,
             random = ~ genotype,
             family = "gaussian",
             prior = prior1,
             nitt = 1300000, burnin = 300000, thin = 1000,
             data = data1,
             pr=T,pl=T)
```

Model 2 is fitted to the complete data set. It tests the effects of reproductive mode, parasite year and the interaction on the proportion of hosts infected. Host genotype and family are fitted as random effects. See below for the R code for the prior and model.

```
prior2 <- list(R=list(V=diag(1)*1, nu=0.002),
               G=list(G1=list(V=diag(4), nu=4,
 alpha.mu=rep(0,4), alpha.V=diag(4)*1000),
               G2=list(V=1,nu=1,alpha.mu=0,alpha.V=1000)))

m2<-MCMCglmm(logit(p.inf) ~ rmode + parayear + rmode:parayear,
             random = ~ us(parayear:rmode):fam + genotype,
             family = "gaussian",
             prior = prior2,
             nitt = 1300000, burnin = 300000, thin = 250,
             data = data1,
             pr=T,pl=T)
```

Model 3 is also fitted to the complete data set and has the same fixed and random effects structure as model 2. It tests the effects of reproductive mode, parasite year

and the interaction on the density of spores within infected hosts. Host genotype and family are fitted as random effects. See below for the R code for the model (the prior is the same as the one used in model 2).

```
M3<-MCMCglmm(as.integer(spores) ~ rmode + parayear +
 rmode:parayear,
                 random = ~ us(parayear:rmode):fam + genotype,
                 family = "poisson",
                 prior = prior2,
                 nitt = 1300000, burnin = 300000, thin = 250,
                 data = spd1,
                 pr=T,pl=T)
```

Table S1. Summary statistics testing the effects of host reproductive mode and parasite year on: **A** the proportion of infected hosts and **B** the densities of spores per infected host. The parameter posterior modes and 95% credible intervals [CI] are shown for each factor in the two models.

	Posterior mode	Lower 95% CI	Upper 95% CI	Effective sample size	P_{MCMC}
**A** Proportion of infected hosts					
Intercept	-0.18	-0.79	0.48	4000	0.57
Reproductive mode	-0.44	-1.27	0.08	4000	0.09
Parasite year	0.03	-0.6	0.65	4000	0.91
R. mode x P. year	-1.13	-1.77	-0.29	4000	0.014
**B** Parasite spore densities					
Intercept	13.35	13.07	13.59	4000	<0.0001
Reproductive mode	0.05	-0.31	0.43	4000	0.82
Parasite year	0.39	0.13	0.63	4000	0.002
R. mode x P. year	-0.33	-0.7	0.03	4000	0.069
